# Persistent neuroinflammation and cognitive impairment in a rat model of acute diisopropylfluorophosphate intoxication

**DOI:** 10.1186/s12974-016-0744-y

**Published:** 2016-10-12

**Authors:** Brenna M. Flannery, Donald A. Bruun, Douglas J. Rowland, Christopher N. Banks, Adam T. Austin, David L. Kukis, Yonggang Li, Byron D. Ford, Daniel J. Tancredi, Jill L. Silverman, Simon R. Cherry, Pamela J. Lein

**Affiliations:** 1 Department of Molecular Biosciences, School of Veterinary Medicine, University of California-Davis, Davis, CA 95616 USA; 2 Center for Molecular and Genomic Imaging, University of California-Davis, Davis, CA 95616 USA; 3 Department of Pediatrics, University of California-Davis Medical Center, Sacramento, CA 95817 USA; 4 Division of Biomedical Sciences, University of California-Riverside School of Medicine, Riverside, CA 92521 USA; 5 Department of Psychiatry and Behavioral Sciences, University of California-Davis Medical Center, Sacramento, CA 95817 USA; 6 Current address: Office of Environmental Health Hazard Assessment, California Environmental Protection Agency, Sacramento, CA 95812 USA; 7 Current address: Navistar, Inc., 2701 Navistar Drive, Lisle, IL 60532 USA

**Keywords:** Cognitive deficits, Diisopropylfluorophosphate, Neurodegeneration, Neuroinflammation, Organophosphate neurotoxicity, PET imaging, Sublethal effects, TSPO

## Abstract

**Background:**

Acute intoxication with organophosphorus (OP) cholinesterase inhibitors can trigger convulsions that progress to life-threatening status epilepticus. Survivors face long-term morbidity including mild-to-severe decline in memory. It is posited that neuroinflammation plays a key role in the pathogenesis of OP-induced neuropsychiatric deficits. Rigorous testing of this hypothesis requires preclinical models that recapitulate relevant phenotypic outcomes. Here, we describe a rat model of acute intoxication with the OP diisopropylfluorophosphate (DFP) that exhibits persistent neuroinflammation and cognitive impairment.

**Methods:**

Neuroinflammation, neurodegeneration, and cognitive function were compared in adult male Sprague Dawley rats injected with an acutely toxic dose of DFP vs. vehicle controls at multiple time points up to 36 days post-exposure. Neuroinflammation was quantified using immunohistochemical biomarkers of microglia (ionized calcium-binding adapter molecule 1, IBA1) and activated astrocytes (glial fibrillary acidic protein, GFAP) and positron emission tomography (PET) imaging of [^11^C]-(R)-PK11195, a ligand for the 18-kDa mitochondrial membrane translocator protein (TSPO). FluoroJade-B staining was used to assess neurodegeneration; Pavlovian conditioning, to assess cognitive function.

**Results:**

Animals exhibited moderate-to-severe seizures within minutes of DFP injection that continued for up to 6 h post-injection. As indicated by IBA1 and GFAP immunoreactivity and by PET imaging of TSPO, acute DFP intoxication triggered neuroinflammation in the hippocampus and cortex during the first 3 days that peaked at 7 days and persisted to 21 days post-exposure in most animals. Neurodegeneration was detected in multiple brain regions from 1 to 14 days post-exposure. All DFP-intoxicated animals exhibited significant deficits in contextual fear conditioning at 9 and 20 days post-exposure compared to vehicle controls. Whole-brain TSPO labeling positively correlated with seizure severity score, but did not correlate with performance in the contextual fear-conditioning task.

**Conclusions:**

We describe a preclinical model in which acute DFP intoxication causes seizures, persistent neuroinflammation, neurodegeneration, and memory impairment. The extent of the neuroinflammatory response is influenced by seizure severity. However, the observation that a subset of animals with moderate seizures and minimal TSPO labeling exhibited cognitive deficits comparable to those of animals with severe seizures and significant TSPO labeling suggests that DFP may impair learning and memory circuitry via mechanisms independent of seizures or neuroinflammation.

## Background

Organophosphorus (OP) nerve agents and pesticides that inhibit acetylcholinesterase represent a major public health concern [1–3]. Acute intoxication with these OPs can trigger convulsions that progress to life-threatening status epilepticus (SE), and survivors face long-term morbidity, including mild-to-severe cognitive deficits, affective disorders, and recurrent seizures [2–6]. Each year, an estimated 300,000 people die, and many more experience significant morbidity, as a result of suicidal and accidental exposures to OP pesticides [1, 6–8], and there are growing concerns of civilian mass causalities resulting from terrorist use of OP nerve agents or OP pesticides [2, 3, 9, 10]. Current medical countermeasures for OP-induced SE (atropine, oxime, and high-dose benzodiazepine) can terminate seizures and reduce mortality; however, they do so with significant side effects and are effective in protecting against long-term morbidity only if administered within minutes of exposure [2, 5, 7, 8, 11]. These sobering facts underscore the need for improved therapeutic approaches to mitigate the long-term neurological sequelae of acute OP intoxication.

The search for more effective therapeutic strategies has been complicated by uncertainty regarding the pathogenic mechanisms linking acute OP intoxication to persistent neurological deficits. One postulated mechanism is neuroinflammation [4, 12, 13]. Much of the evidence in support of this hypothesis derives from experimental studies of OP nerve agents (reviewed in [4, 12]; see also [14, 15]). Following OP nerve agent-induced SE, there is a rapid and sustained neuroinflammatory response marked by activation of microglia and astrocytes [16–18] and significantly increased brain levels of proinflammatory mediators, including arachidonic acid metabolites [18, 19] and cytokines [20–25]. These neuroinflammatory responses typically coincide with neurodegeneration and either precede or overlap with significant behavioral deficits and recurrent seizures (reviewed in [3–5, 12, 13]). However, whether neuroinflammation is causally linked to long-term neurological deficits has yet to be established in these models.

It is also unknown whether preclinical models of acute intoxication with OP nerve agents are predictive of effects following acute intoxication with OP pesticides. Although all seizure-inducing OPs are potent cholinesterase inhibitors, each elicits a unique profile of toxic effects [26–28], inflammatory responses vary between OP nerve agents and OP pesticides [29–31], and current therapeutic strategies are not equally effective against different OPs [7, 28]. Recent research efforts to address this question have focused on diisopropylfluorophosphate (DFP), an OP pesticide considered to be a credible chemical threat agent (Jessica Cox, Department of Homeland Security, personal communication). Using a rat model of acute DFP intoxication that induces robust seizure behavior, we recently reported significant activation of microglia and astrocytes and upregulation of proinflammatory genes in multiple brain regions at 24 h post-exposure [32], which are still evident at 48 to 96 h post-exposure [31, 33, 34]. These and studies from other groups [35–37] have also documented neurodegeneration over this same post-exposure time frame following acute DFP intoxication. Behavioral studies of acute intoxication with DFP report spatial learning deficits at 4 weeks post-exposure in the Morris water maze [38], impairments at 6 to 12 weeks post-exposure in the novel object recognition task [39], and higher scores on depression-relevant immobility behavior at 8 and 29 days post-exposure [40]. However, to date, there are no data documenting long-term effects on neuroinflammation and functional outcomes in the same animals following acute intoxication with DFP or any other seizure-inducing OP pesticide. These data are critical for assessing the biological plausibility of a causal link between neuroinflammation and persistent neurological deficits and for informing mechanistic studies to rigorously test this hypothesis.

The major goal of this study was to address this data gap by characterizing long-term effects of acute DFP-induced SE on neuroinflammation and behavior. The behavioral readout we chose for these studies was learning and memory, because deficits in this domain have been documented in humans who survive acute OP intoxication [3, 5] and in experimental models of acute intoxication by DFP [38, 39] and other seizure-inducing OPs [4]. Neuroinflammation was quantified in the hippocampus and frontal cortex because learning and memory are heavily dependent on the function of these brain regions [41], and both the hippocampus and cortex are known to be adversely affected by OP nerve agents [42]. In addition, we evaluated positron emission tomography (PET) imaging of the 18-kDa mitochondrial translocator protein (TSPO) as a non-invasive approach for longitudinal quantification of neuroinflammation. TSPO is typically expressed at low levels on the outer mitochondrial membrane of glial cells in the brain; however, upon activation, TSPO expression is significantly upregulated, making it a useful biomarker of activated microglia and astrocytes [43, 44]. Using these approaches, we were able to demonstrate persistent neuroinflammation and impaired cognition in animals acutely intoxicated with DFP.

## Methods

### Animals and DFP exposure

Animals were maintained in facilities fully accredited by the Association for Assessment and Accreditation of Laboratory Animal Care, and all studies were performed with regard for alleviation of pain and suffering under protocols approved by the UC Davis Institutional Animal Care and Use Committee. Adult male Sprague Dawley rats (250–280 g; Charles River Laboratories, Hollister, CA) were housed individually in standard plastic cages under controlled environmental conditions (22 ± 2 °C, 40–50 % humidity) with a normal 12-h light/dark cycle. Food and water were provided ad libitum. All animals were allowed to acclimate for 7 days prior to the initiation of experiments.

**Fig. 1:**
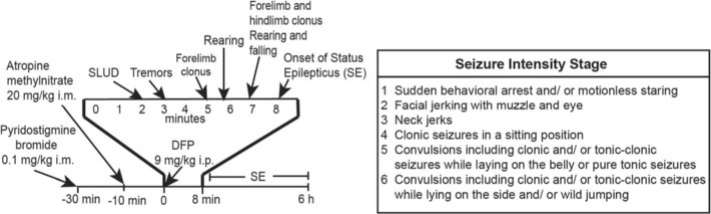
Schematic representation of the rat model of acute DFP intoxication. Adult male Sprague Dawley rats are administered pyridostigmine bromide and atropine methyl nitrate prior to administration of DFP to increase survival. Parasympathomimetic symptoms (SLUD) appear within minutes after injection of DFP and rapidly progress to seizures, with status epilepticus beginning within 10 min after DFP exposure. Seizure behavior is monitored for 6 h post-injection and seizure severity scored from 1 (least severe) to 6 (most severe) as indicated in the figure. *SLUD* salivation, lacrimation, urination, and defecation

Subject rats were injected intraperitoneally (ip) with DFP (Sigma Chemical Company, St Louis, MO) at 9 mg/kg in a total injection volume of 300 μl. In all studies, DFP was diluted with sterile saline within 5 min of administration. To increase survival following DFP exposure, animals were injected intramuscularly (im) with 0.1 mg/kg pyridostigmine bromide (TCI America, Portland, OR) in saline and 20 mg/kg atropine methyl nitrate (TCI America) in saline, 30 and 10 min prior to DFP injection, respectively (Fig. 1). These drugs are centrally inactive [45] but effectively block peripheral cholinergic toxicity, thereby reducing mortality [35]. Vehicle control subjects (VEH) were injected ip with 300 μl saline in place of DFP but were similarly pretreated with pyridostigmine (0.1 mg/kg, im) and atropine (20 mg/kg, im). Seizure behavior was monitored for 6 h following DFP injection using a modified Racine scale (Fig. 1), as previously described [46]. Once returned to their home cages, rats were provided DietGel Recovery (ClearH_2_O, Portland, ME) for 3–5 days or until they resumed consumption of solid chow.

### Histological analyses

For histological analyses, a total of 9 VEH and 23 DFP rats were dosed; 2–3 VEH animals were euthanized at 1, 3, 7, and 21 day post-injection; 3–7 DFP animals were euthanized at 1, 2, 3, 7, 14, and 21 days post-injection. Brains were harvested from animals deeply anesthetized with 5 % isoflurane in oxygen and subsequently perfused transcardially with cold PBS followed by cold 4 % paraformaldehyde solution in phosphate-buffered saline (PBS; pH 7.2). The brains were post-fixed with 4 % paraformaldehyde at 4 °C for 24 h and then transferred to 30 % sucrose solution in PBS. Upon removal from the 30 % sucrose solution, the brains were laterally bisected, frozen in O.C.T. compound (Sakura Finetek, Torrance, CA) on dry ice, and then sectioned on a cryostat (Microm HM 505E, Thermo Fisher, Waltham, MA) to generate 10-μm sagittal sections. Sections were stored at –80 °C until further processed.

To detect neuronal cell damage, sections were labeled with FluoroJade-B (FJB) solution (Chemicon International, Temecula, CA), as previously described [35]. To assess astrogliosis and microglial cell activation, sections were immunostained for glial fibrillary acidic protein (GFAP, 1:500 dilution; Dako, Glostrup, Denmark) or ionized calcium-binding adapter molecule 1 (IBA1, 1:1000 dilution; Wako Chemicals, Richmond, VA), respectively, as previously described [47]. GFAP and IBA1 immunoreactivity was visualized by confocal microscopy and quantified using image analysis software (MetaMorph, Molecular Devices, Sunnyvale, CA), as previously described [47]. Individuals blinded to the treatment condition determined the number of activated vs. resting IBA1 immunopositive microglia according to published morphologic criteria [48]. Activated and resting microglia were quantified across the entire hippocampus and along the entire dorsal boundary of the cortex to an inward depth of 500 μm from the dorsal edge. All endpoints were examined in at least three serial sections per animal, and data were collected from the same level of the brain across all animals.

### In vivo imaging

The radiosynthesis of the TSPO ligand [^11^C]-(R)-PK11195 was adapted from a previously published method [49]. Briefly, cyclotron (Siemens RDS 111)-produced [^11^C]CO_2_ was converted to [^11^C]CH_3_I in the gas phase (GE Tracerlab FX-C Pro) and bubbled into a solution of (R)-N-desmethyl-PK11195 (1 mg) and NaH (1–2 mg) in DMSO (250 μl). The reaction was quenched in H_2_O (1.5 ml) and the labeled product purified by semi-preparative high-performance liquid chromatography on a C-18 column eluted in 70 % ethanol (aq). The pure product was collected in a vial containing Tween-80 (Sigma) and then dried (110 °C) before formulation with PBS. Tween-80 (final concentration, 0.6 % *v*/*v*) was used to prevent adsorption of [^11^C]-(R)-PK11195 onto plastic vials, syringes, and extension tubing. Twenty-four syntheses were performed with a decay-corrected yield of 12.7 ± 2.7 % (mean ± SD) and radiochemical purity of >95 %. The mean specific activity at the end of the formulation was 2160 ± 1370 Ci/mmol.

Rats (*n* = 3 VEH and 6 DFP) were imaged using PET prior to DFP injection to collect baseline TSPO labeling and then imaged again at 2, 7, and 21 days post-DFP intoxication. Animals were anesthetized with approximately 2 % isoflurane in oxygen gas and maintained on 1–2 % isoflurane. Approximately 1.0 mCi of [^11^C]-(R)-PK11195 ligand was injected via a tail vein catheter. Animals were then transferred to the animal bed for scanning. Two animals were secured onto an imaging bed in a cheek-to-cheek position, with the middle of the brains centered in the axial field of view. Twenty-minute emission scans began 10 min post-injection of [^11^C]-(R)-PK11195. Images were acquired with a Focus 120 Small Animal PET Scanner (Siemens Preclinical Solutions, Knoxville, TN) with a resolution of 1.3 mm. Animals were kept warm with heating lamps throughout the imaging session.

A single magnetic resonance imaging (MRI) scan was performed 7–14 days post-DFP intoxication at either the UC Davis Nuclear Magnetic Resonance (NMR) facility on a Bruker Biospec 7 tesla scanner operating Paravision v 4.0 software or at the UC Davis Center for Molecular and Genomic Imaging on a Bruker Biospec 7 tesla magnet operating Paravision v 5.1. Rodent brain imaging was optimized at the NMR facility using a T2-weighted fast spin echo (FSE) sequence with TE = 30 ms, TR = 3350 ms, 0.2 mm in-plane resolution (256 × 256 matrix) zero filled once, and 0.7-mm slice thickness. FSE scans at the CMGI facility were acquired with TE = 28 ms, TR = 4250 ms, 0.137 mm in-plane resolution (256 × 256 matrix, zero filled once), and 0.5-mm slice thickness. Scan times on both systems were approximately 36 min.

Images were analyzed using Inveon Research Workplace (Siemens Preclinical Solutions) software. Both the PET and MR images were imported and co-registered for analysis. Regions of interest for analysis were annotated on the MR image and included the whole brain, rostral and caudal hippocampus, amygdala, lateral dorsal thalamus, medial dorsal thalamus, piriform cortex, somatosensory cortex, septum, and reuniens nucleus.

### Behavioral testing

Learning and memory were assessed using contextual fear conditioning as previously described [41]. Animals were trained for conditioned stimulus (CS)-unconditioned stimulus (UCS) associations using two identical operant chambers (Med Associates, St. Albans, VT) that were calibrated to deliver foot shocks at 0.5 or 0.7 mA. Each chamber was 30.5 cm × 24.1 cm × 21 cm, the side walls were aluminum, the front wall was clear polycarbonate, the back wall and ceiling were opaque Plexiglas, and the floor was composed of stainless steel rods placed 1.6 cm apart. A scrambled foot shock was delivered through the floor. Each conditioning chamber was surrounded by a sound-attenuating environmental chamber and equipped with an overhead CCTV to record behavior. Chambers were thoroughly cleaned with 70 % ethanol between subjects.

To condition animals, subjects were placed in a house-lit chamber for 2 min, followed by presentation of an auditory CS, 78-dB white noise for 30 s, followed by the UCS, a 2-s foot shock delivered through the floor grid. Two separate cohorts of animals were tested. One cohort (*n* = 6 VEH and 6 DFP) received three consecutive foot shocks at 0.7 mA each with 30 s of silence between the foot shocks; the second cohort (*n* = 10 VEH and 16 DFP) received a single foot shock at 0.5 mA. An olfactory cue was added before fear conditioning by dabbing a drop of lemon dish soap solution on the metal tray beneath the grid floor. After the CS-US pairing, subjects were left in the chamber for another 1 min, during which time behavior was recorded by a CCTV. Subjects were then returned to their home cage for 24 h. The next day, memory of the fear-associated context was quantified by placing subjects in the same operant chamber with identical visual, floor-texture, and olfactory cues as the day before during tone-shock pairing. Each animal was placed in the chamber for 5 min, and its behavior recorded in the absence of the auditory CS and the US foot shock. The rat was then returned to its home cage. The dependent variable for assessing fear conditioning was freezing, defined as the absence of any movement except that needed for respiration. Freezing was manually scored from videos at 8-s intervals by two independent observers blinded to treatment.

The open-field test was used to assess locomotion in a novel open arena in a separate cohort of animals. Subjects (*n* = 5 per group) were placed in a black matte box made of medium-density fiberboard (MDF) measuring 50 cm × 50 cm × 45 cm under 65-lx lighting and in 73 °F room temperature. The total distance traveled by each subject was tracked for 15 min using center point tracking (Ethovision XT9 system, Noldus Information Technology, Leesburg, VA). Detection settings were set to static subtraction with video sampling at 10 frames per second. Upon completion of the task, subjects were returned to their home cages and the open-field apparatus cleaned with 70 % ethanol before the next subject. Ethovision software was used to calculate the total distance traveled during the 15-min testing session.

### Statistics

The units of statistical analysis for each outcome were clustered, with observations from each animal being taken at multiple regions of the brain, at multiple days, or both. Hence, we used statistical analysis methods for clustered data to increase the efficiency and robustness of our analysis by accounting for within-cluster correlations. Statistical analysis began with graphical representation of study data to identify patterns of central tendency and variation and to suggest data transformations and model specifications that would improve the validity of statistical inferences.

Comparisons among treatment conditions in mean levels of study outcomes were estimated and tested using generalized linear models for clustered data in Version 12 of Stata (College Station, TX), using appropriate link and variance functions according to the distribution of the outcome. Behavioral data were expressed as proportion time freezing. These were analyzed using logistic regression techniques for clustered survey data. Logarithmic transformations were applied to select count and area measurements to stabilize the variances for purposes of comparing and to allow the use of mixed-effects linear regression techniques on the log-transformed data. Otherwise, mixed-effects Poisson regression models were applied.

Graphical depictions of immunohistochemical outcomes revealed that there was little variation among observations from vehicle animals. Similarly, at day 21, observations in DFP animals exhibited little to no variability. Hence, for immunohistochemical outcomes, vehicle animals were pooled across multiple days and used as the reference group for day-specific DFP groups, with day 21 DFP data dropped from the analysis. The treatment group factor for the IHC outcomes thus had the following levels: vehicle vs. day 1 DFP vs. day 2 DFP vs. day 3 DFP vs. day 7 DFP vs. day 14 DFP. For these outcomes, mixed-effects models were fit with the data to allow brain region-specific comparisons of day-specific DFP groups vs. the vehicle control, using main effects terms for the treatment group and the brain region along with an interaction term for the treatment group X brain region. These latter terms were used in Wald tests to assess between-region heterogeneity in pairwise comparisons among the treatment group factor levels. In case none was found, the model was simplified to a main effects only model.

For behavioral and TSPO imaging data, mixed-effects regression models were used to estimate day-specific comparisons between DFP and vehicle controls. Wald tests for interaction were used similarly as above to assess heterogeneity in DFP vs. vehicle contrasts by either brain region (in the TSPO imaging data) or by day of observation (for behavioral data). A two-way analysis of variance (ANOVA) with Holm-Sidak multiple comparisons was used to assess differences in percent freezing during context testing in DFP vs. vehicle rats over time. Two-tailed Student’s *t* test was performed to compare the locomotor activity of DFP and vehicle rats in the open-field test.

## Results

### Acute DFP intoxication causes persistent neuronal damage and neuroinflammation

**Fig. 2:**
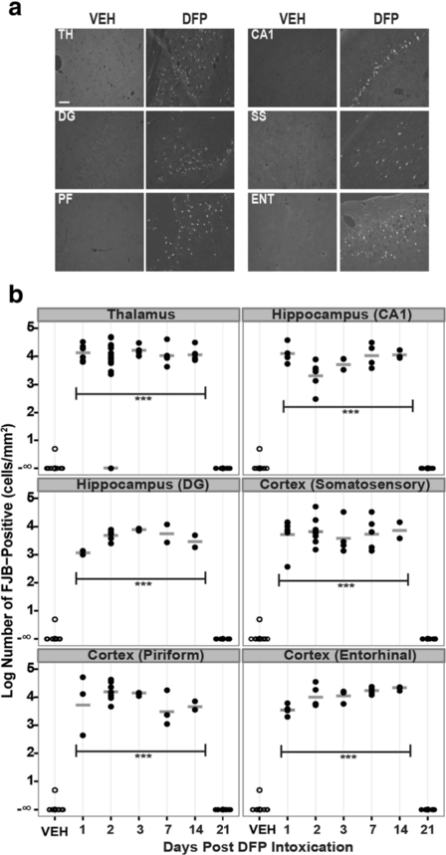
Acute DFP intoxication causes persistent neuronal degeneration in multiple brain regions. Rats injected with DFP (9 mg/kg ip) or an equal volume (300 μl) of vehicle (VEH) were euthanized at 1, 2, 3, 7, 14, and 21 days post-exposure to collect the brains for FluoroJade-B (FJB) staining. **a** Representative photomicrographs of FJB labeling in the thalamus (*TH*), CA1 region (*CA1*), and dentate gyrus (*DG*) of the hippocampus, as well as somatosensory (*SS*), piriform (*PF*), and entorhinal (*ENT*) cortices of the VEH and DFP-exposed rats at 7 days post-injection. *Bar* = 50 μm. **b** Dot plots of the log number of FJB-labeled cells in specific brain regions. Each *dot* represents the data from an individual animal, while the *horizontal gray bars* represent the geometric mean of FJB-labeled cells in that brain region at each time point. Note: No FJB-labeled cells were detected in any brain region of any VEH animal at any time point or in any DFP-exposed animals at 21 day post-exposure; similarly, no FJB-labeled cells were detected in the thalamus of one DFP-exposed animal at 2 days post-exposure, resulting in a geometric mean of negative infinity for these conditions. ***Significantly different from VEH at *p* < .001

Consistent with previous studies from our group [35] and others [33, 36], ip injection with DFP at 9 mg/kg following pretreatment with pyridostigmine and atropine methyl nitrate (Fig. 1) caused severe seizure activity in adult male Sprague Dawley rats in the absence of significant mortality. To extend previous analyses of neuronal cell injury up to 72 h following acute DFP intoxication [35], FJB-labeled cells were quantified in multiple brain regions of animals injected with a single dose of DFP or saline (VEH) at 1, 2, 3, 7, 14, and 21 days post-injection. Across all brain regions and time points examined, FJB labeling was not detected in the brain sections from VEH animals (Fig. 2). In contrast, in DFP animals, significant numbers of FJB-labeled cells were observed at 1, 2, 3, 7, and 14 days post-exposure in the thalamus, CA1, and dentate gyrus of the hippocampus and multiple regions within the cortex (Fig. 2), and these effects were bilaterally symmetrical. However, in all of these brain regions, FJB labeling returned to levels comparable to those observed in VEH animals by 21 days post-DFP injection. At no time point were FJB-labeled cells detected in the cerebellum of DFP animals (data not shown).

**Fig. 3:**
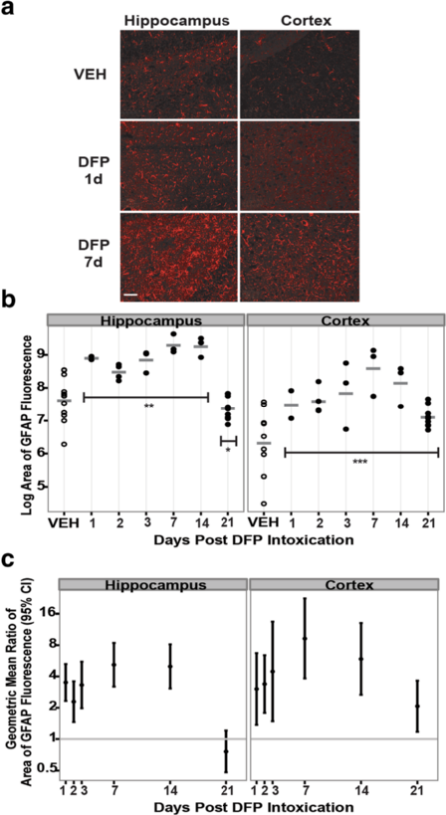
Acute DFP intoxication causes persistent astrogliosis in the hippocampus and cortex. Rats injected with DFP (9 mg/kg ip) or an equal volume (300 μl) of vehicle (VEH) were euthanized at 1, 2, 3, 7, 14, and 21 days post-exposure to collect the brains for GFAP immunostaining. The area of GFAP immunoreactivity per field (three fields per section in three sections per brain) was quantified in the cortex and hippocampus as a measure of astrogliosis. **a** Representative images of GFAP immunoreactivity in the hippocampus and cortex of rats administered VEH or DFP at 1 and 7 days post-injection. *Bar* = 50 μm. **b** Dot plots of the log area of GFAP immunofluorescence in the hippocampus and cortex, illustrating individual variation in GFAP immunoreactivity within and between groups. Each *dot* represents the data from an individual animal, while the *horizontal gray bars* represent the geometric mean of the log area of GFAP immunofluorescence for all animals at each time point. Note: VEH across all time points are shown as one group because there were no significant time-dependent differences between them. *Significantly different from VEH at *p* < .05; **p < .01; ***p < .001. **c** Variation between experimental groups is illustrated as the geometric mean ratio (*dot*) and 95 % confidence interval (*bar*) of the area of GFAP immunofluorescence in DFP-exposed rats relative to VEH controls. Confidence intervals not overlapping *y* = 1 (*gray horizontal bar*) indicate a significant difference between DFP and VEH conditions (*p* < .05)

To assess neuroinflammatory responses to acute DFP intoxication, brain sections were immunostained for GFAP, a marker of reactive astrocytes, and IBA1, a marker of microglia. In both brain regions, reactive astrogliosis and microglial activation were bilaterally symmetrical. As illustrated in representative photomicrographs (Fig. 3a), relative to time- and region-matched samples from VEH controls, acute intoxication with DFP appeared to increase GFAP immunoreactivity at 1 and 7 days post-exposure in the hippocampus and cortex. Quantification of GFAP immunoreactivity in these brain regions indicated that DFP did not change the intensity of GFAP immunofluorescence in either the hippocampus or cortex (data not shown), but rather, DFP significantly increased the area of GFAP immunoreactivity in both brain regions (Fig. 3a, b). In the hippocampus, DFP significantly increased the area of GFAP immunoreactivity from 1 to 14 days post-injection with peak labeling at 7 and 14 days post-injection. At 21 days post-exposure, the area of GFAP immunoreactivity in the hippocampus of DFP animals was significantly less than that in VEH animals. DFP similarly increased the area of GFAP immunoreactivity in the cortex at 1 day post-exposure; however, unlike the hippocampus, the area of GFAP immunoreactivity in the cortex remained significantly increased at 21 days, with the maximal level observed at 7 days post-exposure.

**Fig. 4:**
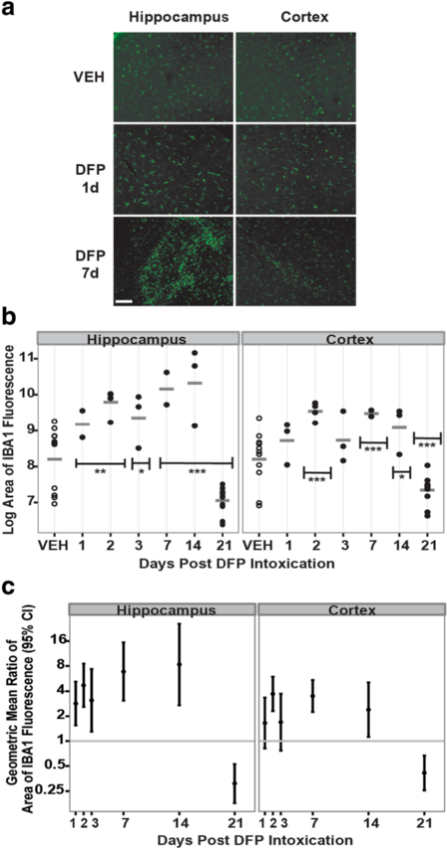
Acute DFP intoxication increases expression of the microglial biomarker IBA1 in the hippocampus and cortex. Rats injected with DFP (9 mg/kg ip) or an equal volume (300 μl) of vehicle (VEH) were euthanized at 1, 2, 3, 7, 14, and 21 days post-exposure to collect the brains for IBA1 immunostaining. The area of IBA1 immunoreactivity per field (three fields per section in three sections per brain) was quantified in the cortex and hippocampus to assess microglial activation. **a** Representative photomicrographs of IBA1 immunoreactivity in the hippocampus and cortex of rats administered VEH or DFP at 1 and 7 days post-injection. *Bar* = 50 μm. **b** Dot plots of the log area of IBA1 immunofluorescence in the hippocampus and cortex. Each *dot* represents the data from an individual animal while the *horizontal gray bars* represent the geometric mean of the log area of IBA1 immunofluorescence for all animals at each time point. Note: VEH across all time points are shown as one group because there were no significant time-dependent differences between them. *Significantly different from VEH at *p* < .05; **p < .01; ***p < .001. **c** The geometric mean ratio (*dot*) and 95 % confidence interval (*bar*) of the area of IBA1 immunofluorescence in DFP-exposed rats relative to VEH controls. Confidence intervals not overlapping *y* = 1 (*gray horizontal bar*) indicate a significant difference between DFP and VEH conditions (*p* < .05)

**Fig. 5:**
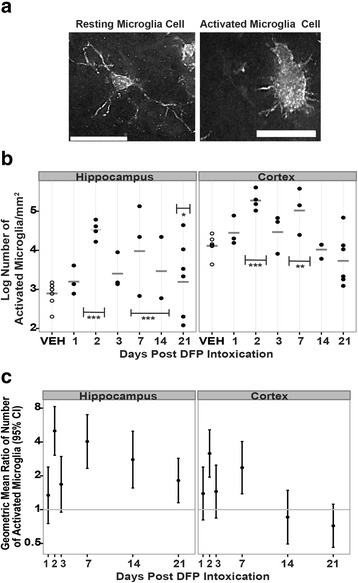
Acute DFP intoxication increases the number of activated microglia in the hippocampus and cortex. **a** Representative photomicrographs illustrating the morphology of resting vs. activated IBA1 immunopositive microglia. *Bar* = 25 μm. **b** Dot plots illustrating the log number of activated microglia per mm^2^ in the hippocampus and cortex of VEH and DFP-exposed animals as a function of days post-exposure. Each *dot* represents the data from an individual animal, while the *horizontal gray bars* represent the geometric mean for all brain sections at each time point. Note: VEH across all time points are shown as one group because there were no significant time-dependent differences between them. *Significantly different from VEH at *p* < .05; **p < .01; ***p < .001. **c** Geometric mean ratio (*dot*) and 95 % confidence interval (*bar*) of the number of activated microglia in DFP-exposed rats relative to VEH. Confidence intervals not overlapping *y* = 1 (*gray horizontal bar*) indicate a significant difference relative to VEH (*p* < .05)

To further assess DFP-induced neuroinflammation, the number and activation state of microglial cells were evaluated. The area of IBA1 immunoreactivity was used as an indicator of microglia number (Fig. 4), while microglial cell morphology was used to assess the activation state of IBA1 immunopositive cells (Fig. 5). As shown in representative micrographs (Fig. 4a), acute DFP intoxication appeared to increase IBA1 immunoreactivity in both the hippocampus and cortex at 1 and 7 days post-injection. Quantification of IBA1 immunoreactivity in these brain regions indicated that DFP did not change the intensity of IBA1 immunofluorescence in either the hippocampus or cortex (data not shown); however, DFP increased the area of IBA1 immunoreactivity in both brain regions. In the hippocampus, the area of IBA1 immunoreactivity was significantly increased in DFP relative to VEH animals at days 1 through 14 post-exposure; however, at 21 days post-exposure, the area of IBA1 immunoreactivity in the hippocampus was significantly decreased in DFP animals relative to VEH controls (Fig. 4b, c). In the cortex, DFP significantly increased the area of IBA1 immunoreactivity at 2, 7, and 14 days post-exposure, but not at 1 or 3 days post-exposure (Fig. 4b, c). At 21 days post-exposure, DFP caused a significant decrease in IBA1 immunoreactivity relative to VEH. Using morphological criteria to determine the activation state of microglia (Fig. 5a), DFP was observed to cause multiple waves of microglial activation in the hippocampus and cortex as evidenced by a significant increase at 2 days, followed by a decrease to VEH levels at 3 days, followed by a significant increase again at 7 days post-exposure (Fig. 5b, c). In the hippocampus, the second wave of microglia activation persisted to 21 days post-exposure whereas in the cortex, the number of activated microglia returned to VEH levels by 14 days post-exposure (Fig. 5b, c).

### PET imaging of translocator protein 18 kDa (TSPO) demonstrates delayed and persistent neuroinflammation in DFP animals

**Fig. 6:**
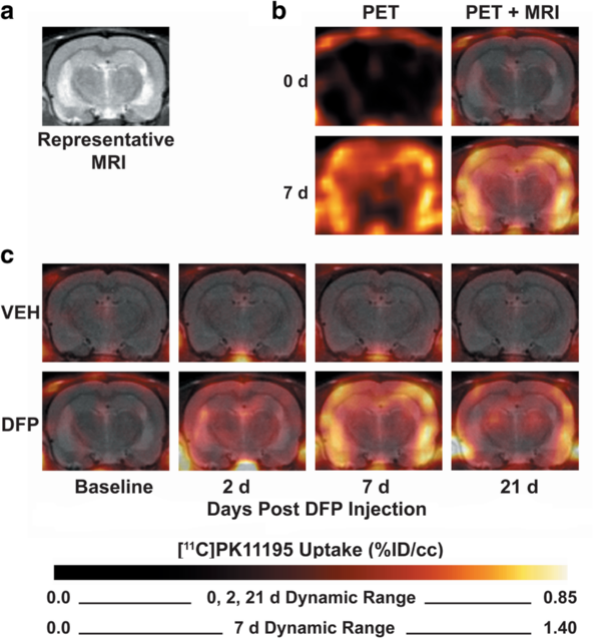
TSPO PET imaging detects neuroinflammation in DFP-intoxicated rats. TSPO expression was quantified in rats injected with the TSPO ligand [^11^C]-PK11195. All animals were imaged before administration of VEH or DFP to collect baseline TSPO labeling; the same animals were imaged again at 2, 7, and 21 days post-injection with VEH or DFP. **a** Representative magnetic resonance image (MRI) was used for anatomic registry of PET images. **b** Overlay of MRI and [^11^C]-PK11195 PET data for an animal with typical response at baseline and at 7 days post-DFP intoxication. **c** Longitudinal comparison of [^11^C]-PK11195 uptake in a VEH (*top row*) vs. DFP-intoxicated animal (*bottom row*). Note: For the seventh day images, the upper limit of the *color scale* for PET imaging is increased to reflect a larger dynamic range of [^11^C]-PK11195 uptake values at this time point. *%ID/cc* percent injected dose per cc

PET imaging with the TSPO radioligand [^11^C]-(R)-PK11195 was used as a second approach for assessing neuroinflammation following acute DFP intoxication. Images were obtained at day 0 (baseline) and at 2, 7, and 21 days post-exposure. MR images obtained from the same animals at 7 or 14 days post-exposure were merged with PET images for anatomic registration (Fig. 6a, b). PET images showed that [^11^C]-(R)-PK11195 incorporation in the whole brain of VEH-treated rats was minimal and did not change as a function of time post-injection (Fig. 6c). In contrast, in DFP animals, whole brain [^11^C]-(R)-PK11195 binding was detected at 2 days and reached maximal binding at 7 days post-DFP injection (Fig. 6c). By 21 day post-exposure, [^11^C]-(R)-PK11195 binding was decreased relative to 7 days but was still noticeably above baseline.

**Fig. 7:**
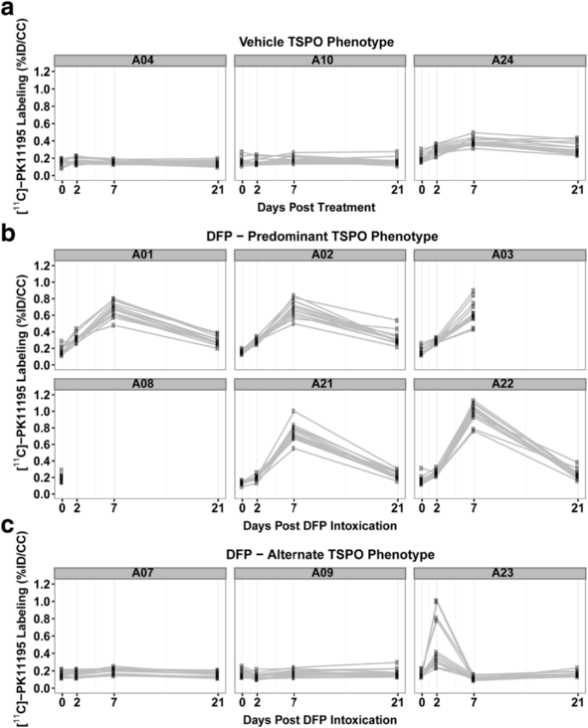
Patterns of TSPO labeling in VEH vs. DFP-intoxicated rats. Rats injected with the TSPO ligand [^11^C]-PK11195 were imaged prior to administration of VEH or DFP (day 0) and again at 2, 7, and 21 days post-exposure. Each *plot* represents an individual rat (with a unique identifier shown at the top of the plot) and each *line* within a plot represents a separate brain region (amygdala, dorsal lateral and medial dorsal thalamus, caudal and rostral hippocampus, piriform cortex, somatosensory cortex, reuniens nucleus, and septum). **a** The TSPO expression pattern for three VEH rats. **b** The predominant TSPO expression pattern imaged in DFP-intoxicated animals (six of nine) showing a predominant peak in TSPO labeling at 7 days post-exposure. Animal A08 died after baseline imaging, within hours after the DFP injection, and animal A03 died several days after the seventh day imaging. **c** Alternate TSPO expression phenotypes observed in three of the nine DFP-exposed rats. *%ID/CC* percent of injected dose per cc

**Fig. 8:**
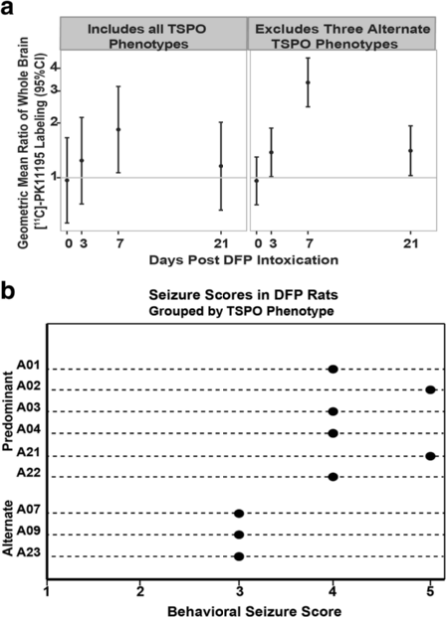
TSPO expression is persistently upregulated in DFP-intoxicated animals that experience more severe seizures. **a** The geometric mean ratio (*dot*) of TSPO labeling with 95 % confidence interval (*bar*) in DFP-exposed rats relative to VEH controls. Confidence intervals not overlapping *y* = 1 (*gray horizontal bar*) indicate a significant difference relative to VEH (*p* < .05). The data presented in the *left panel* include all TSPO phenotypes (*n* = 9 DFP-exposed animals as shown in Fig. 7), while the data presented in the *right panel* exclude the three DFP-intoxicated animals expressing the alternate TSPO phenotypes, as shown in Fig. 7. **b** The predominant TSPO expression phenotype is highly associated with more severe seizure behavior as indicated by higher seizure scores (*p* < .01 as determined using the Wilcoxon signed rank test)

Co-registration of MR and PET images enabled a more accurate volume-of-interest drawing to quantify region-specific changes in [^11^C]-(R)-PK11195 incorporation. With the exception of 1 of 12 animals used in this study, within any given brain, the patterns of change in TSPO labeling over time were similar across multiple brain regions (amygdala, dorsal lateral and medial dorsal thalamus, caudal and rostral hippocampus, piriform cortex, somatosensory cortex, reuniens nucleus, and septum) (Fig. 7). In the one exception, which was a DFP animal (A23), three of the brain regions, the medial dorsal thalamus, reuniens nucleus, and septum, exhibited significantly higher [^11^C]-(R)-PK11195 incorporation than the other brain regions at 2 days post-exposure. Among all VEH animals, there was little increase in [^11^C]-(R)-PK11195 labeling (Fig. 7a) over the 21-day post-exposure period. In contrast, among all the DFP animals, there was heterogeneity in the pattern of TSPO labeling over time. Most DFP animals exhibited a characteristic TSPO-labeling phenotype with maximal [^11^C]-(R)-PK11195 incorporation at 7 days post-exposure (Fig. 7b). Interestingly, three DFP animals exhibited alternate TSPO-labeling phenotypes (Fig. 7c). Two of these showed negligible [^11^C]-(R)-PK11195 labeling over the 21-day post-exposure period while a third showed peak labeling at 2 days post-exposure. Nevertheless, the geometric mean ratio of [^11^C]-(R)-PK11195 incorporation in DFP animals was still significantly increased relative to VEH controls at 7 days even when animals with “alternate” TSPO phenotypes were included in the statistical analyses (Fig. 8a).

To investigate a potential explanation for the alternate TSPO phenotypes observed in a subset of DFP animals, the relationship between [^11^C]-(R)-PK11195 binding and seizure severity was investigated. DFP animals expressing alternate TSPO phenotypes had a lower maximal seizure score relative to those rats displaying the predominant TSPO phenotype (Fig. 8b).

### Acute DFP intoxication leads to persistent learning and memory deficits

**Fig. 9:**
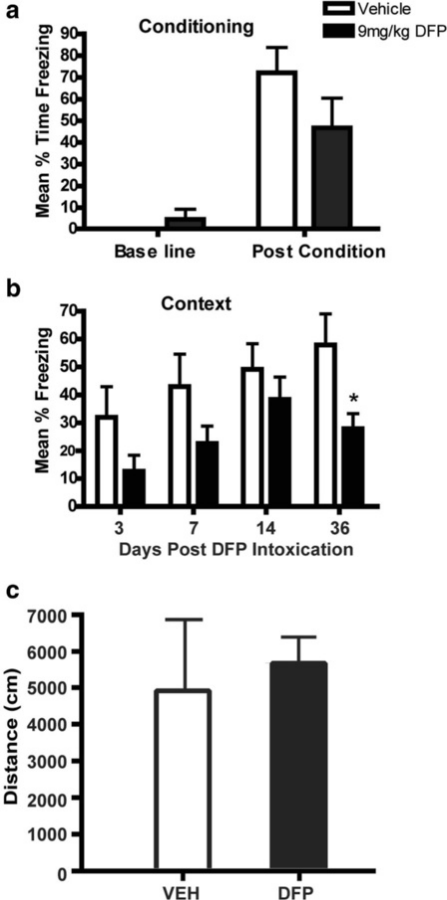
Acute DFP intoxication causes delayed learning and memory deficits without changing locomotor activity. **a** Animals were conditioned 2, 6, 13, and 35 days post-exposure using a three-shock conditioning paradigm with foot shocks at 0.7 mA every 30 s. **b** Animals were tested 24 h later for hippocampal-dependent contextual learning and memory. Data presented as mean ± SD (*N* = 6 per treatment group). *Significantly different from VEH on the same-day post-exposure (*p* < .05) as determined by two-way ANOVA followed by Holm-Sidak post hoc test. **c** Locomotor activity was quantified during a 15-min open-field test at 7 days post-exposure in DFP-intoxicated and vehicle control animals. Data presented as the mean ± SD (*N* = 5 per group). No significant differences between groups were identified using two-tailed Student’s *t* test

Contextual fear conditioning was used to assess the effects of acute DFP intoxication on learning and memory. An initial cohort of animals was tested using a three-shock conditioning paradigm with a 0.75-mA foot shock. Animals were conditioned on days 2, 6, 13, and 35 post-DFP or vehicle exposure. Analysis of baseline and post-conditioning freezing behavior identified no significant differences between VEH and DFP animals (Fig. 9a), indicating that both DFP and VEH animals responded to the CS as expected (F_(1, 12)_ = 66.44, *p* < .001). Analysis of freezing behavior during the context test 24 h later revealed a significant effect of treatment across time (F_(3, 30)_ = 3.3587, *p* < .05). Holm-Sidak post hoc testing indicated that DFP animals froze significantly less than VEH at 36 days post-exposure (Fig. 9b). Testing of these animals in the open-field test at 7 days post-exposure indicated that acute DFP intoxication did not alter locomotor behavior (Fig. 9c), suggesting that the differences in freezing behavior noted in the contextual fear-conditioning test are not due to effects of DFP on locomotor behavior.

**Fig. 10:**
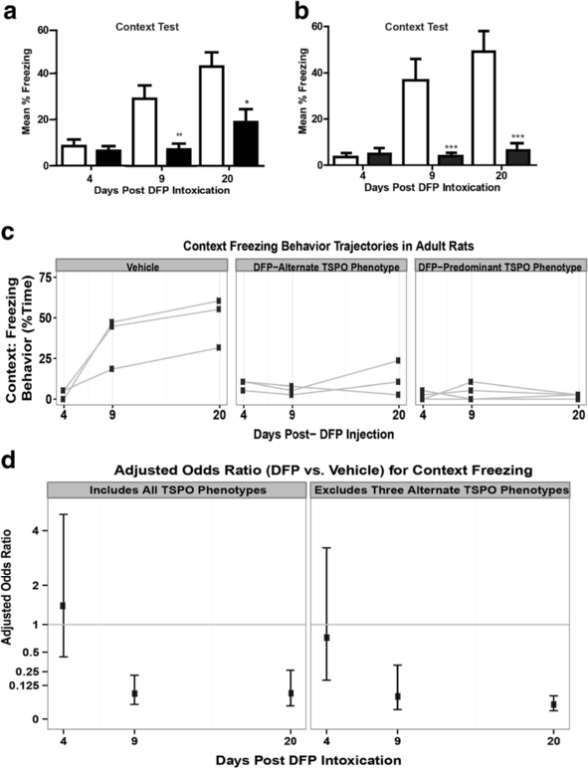
Cognitive deficits are not strongly associated with TSPO labeling. **a** DFP and VEH animals were conditioned 3, 8, and 19 days post-exposure using a single paired shock conditioning paradigm with foot shocks at 0.5 mA and then tested 24 h later for hippocampal-dependent contextual learning and memory (*N* = 10 VEH and 16 DFP-exposed animals). **b** Data from contextual fear conditioning in the subset of animals (*N* = 3 VEH and 6 DFP-exposed animals) also imaged by PET at 2, 7, and 21 days post-exposure. Data presented as the mean ± SD. *Significantly different from VEH on the same-day post-exposure as determined by two-way ANOVA followed by Holm-Sidak post hoc test at *p* < .05; **p < .01; ***p < .001. **c** Patterns of freezing behavior as a function of days post-exposure across different treatments and TSPO phenotypes, as described in Fig. 7. Each *line* represents the data from a single animal. **d** Adjusted odds ratio of freezing behavior in DFP-intoxicated rats relative to VEH with 95 % confidence interval. Confidence intervals not overlapping *y* = 1 (*horizontal gray bar*) indicate a significant difference relative to VEH (*p* < .05)

A second cohort of animals was tested using a single-shock conditioning paradigm. The goal was to determine whether cognitive deficits would manifest at an earlier time post-exposure, which would be desirable for longitudinal monitoring of neuroinflammation by PET imaging of the same animals subjected to behavioral testing. For this second study, DFP and VEH animals were conditioned on days 3, 8, and 19 post-exposure using a single CS-US-paired stimulus with a 0.5-mA foot shock. A subset of these animals was imaged by PET on days 2, 7, and 21. Analysis of freezing behavior during the context test within the entire cohort (imaged and non-imaged animals) revealed a significant effect of treatment (F_(1, 24)_ = 30.41, *p* < .001) and testing (F_(2, 48)_ = 17.93, *p* = .001) and an interaction between treatment and day (F_(2, 48)_ = 10.87, *p* = .0001). Holm-Sidak post hoc testing indicated that DFP animals froze significantly less than VEH at 9 days (*p* < .01) and 20 days (*p* < .05) post-exposure, indicating that DFP animals did not remember the context of the CS-US pairing as well as the VEH controls (Fig. 10a). DFP and VEH animals exhibited similar levels of freezing during the context test at 4 days post-exposure (Fig. 10a). Analysis of the subset of imaged animals also revealed a significant interaction between treatment and day (F_(2,17)_ = 26.69, *p* < .001). Holm-Sidak post hoc testing indicated that DFP animals froze significantly less than VEH at 9 days (*p* < .001) and 20 days (*p* < .001) post-exposure but that there was no difference between the groups at 4 days post-exposure (Fig. 10b). Careful analysis of video recordings from both cohorts of animals by two individuals blinded to the experimental group revealed no evidence of seizure behavior in DFP animals during the CS-US pairing or the context testing.

As an initial assessment of a possible relationship between neuroinflammation and impaired learning and memory, freezing behavior was assessed as a function of TSPO phenotype in the subset of animals that underwent both PET imaging of TSPO and behavioral testing (Fig. 10c). At 4 and 9 days post-exposure, the % time spent freezing by DFP animals was similar between the predominant and alternate TSPO phenotypes. At 20 days post-exposure, the % time spent freezing appeared to be slightly less in the predominant vs. the alternate TSPO phenotype. Logistic regression analyses of context-testing outcomes revealed statistically significant comparisons (vs. VEH controls) in the DFP animals such that VEH controls learned to associate the context with a foot shock over time, while DFP-treated rats did not (Fig. 10d). At 4 days post-exposure, VEH and DFP animals exhibited similar percent freezing during the context test (4-day DFP vs. VEH AOR = 1.42, 95 % CI 0.43–4.7; *p* = .52); however, at 9 and 20 days post-exposure, DFP animals exhibited much less freezing compared to VEH controls (AOR at day 9 = 0.07, 95 % CI 0.02–0.21, *p* < .001; AOR at day 20 = 0.07, 95 % CI 0.02, 0.27, *p* < .001). Excluding DFP animals exhibiting alternate TSPO phenotypes from the statistical analyses did not significantly change the results (Fig. 10d).

## Discussion

Previous studies have demonstrated that in adult male rats, an acute exposure to a seizure-inducing dose of DFP triggers a robust neuroinflammatory response during the first few days post-exposure that coincides spatially and temporally with extensive neurodegeneration in multiple brain regions [31–36, 50]. Deficits in spatial learning [38] or memory [39], as well as depression-relevant behavior [40], have also been reported in the weeks following an acute exposure to DFP at this same dose [39] or doses that cause cholinergic toxicity, but not necessarily seizure behavior [38, 40]. Our findings extend these previous observations by demonstrating that the neuroinflammatory and neurodegenerative responses to acute DFP-induced SE persist beyond the initial few days post-exposure. Additionally, data obtained using PET imaging of the TSPO ligand [^11^C]-(R)-PK11195 to monitor neuroinflammation in the same animal over time indicate a positive correlation between seizure severity and TSPO labeling in the whole brain. However, a strong correlation between these outcomes and DFP-induced deficits in learning and memory was not observed.

Acute intoxication with DFP was observed to cause moderate-to-severe seizure activity in rats, consistent with a previous report demonstrating that this same DFP-dosing paradigm triggers electrographic SE in adult male Sprague Dawley rats [39]. It has been previously reported that intense neuronal activity and seizures [51], including SE induced by OP nerve agents [16–18] or OP pesticides [31–34], rapidly activate astrocytes and microglia. Consistent with these reports, acute DFP intoxication caused a rapid and robust neuroinflammatory response in the hippocampus and cortex comprised of reactive astrogliosis, as evidenced by significantly increased GFAP immunoreactivity, and microglial activation, as determined using morphological criteria to assess the activational status of IBA1 immunopositive cells. In both brain regions, reactive astrogliosis preceded microglial activation, although there was an apparent increase in microglial cell number coincident with initial astrocyte activation. In contrast to previous studies of acute DFP intoxication that terminated at 7 days post-exposure [31] or earlier [32–34, 36], we quantified astrogliosis and microglial cell activation out to 21 days post-exposure. While neuroinflammation persisted in both brain regions beyond 7 days post-exposure, the temporal profile was region dependent. Reactive astrogliosis persisted significantly longer in the cortex relative to the hippocampus, and while a second wave of microglial activation was apparent in both brain regions at 7 days post-exposure, this persisted significantly longer in the hippocampus relative to the cortex. Whether the second wave of apparent microglial cell activation represents microglia and/or monocytes recruited from the periphery remains to be determined. Also unknown at this time is whether the neuroinflammatory response triggered by acute DFP intoxication worsens after 21 days post-exposure, as has been reported following acute intoxication with OP nerve agents [4, 30]. Both questions are the focus of ongoing research.

In general concurrence with immunohistochemical evidence of neuroinflammation, PET imaging of TSPO labeling in the brains of DFP-intoxicated rats indicated that the majority (six of nine animals) exhibited significantly increased neuroinflammation in the hippocampus and cortex relative to baseline pre-exposure values in DFP animals and relative to VEH controls at 2, 7, and 21 days. Peak [^11^C]-(R)-PK11195 binding was observed at 7 days post-exposure. However, a subset of animals (three of nine) exhibited an alternate phenotype in which there was either no significant change in binding of [^11^C]-(R)-PK11195 relative to pre-exposure baseline at any of the imaging times post-exposure or, in the case of one animal, a significant increase in TSPO labeling at 2 days post-exposure that returned to baseline pre-exposure levels by 7 days post-exposure. Evaluation of the maximal seizure score observed among all nine DFP animals during the first 4 h post-DFP injection indicated that the animals with the alternate TSPO phenotype had less severe seizures than animals exhibiting the predominant TSPO phenotype. These data strongly suggest that neuroinflammation is positively correlated with seizure severity. This is consistent with previous observations of a positive correlation between seizure severity and increased expression of neuronal cyclooxygenase 2 in the hippocampus, amygdala, piriform cortex, and thalamus of rats acutely intoxicated with the OP nerve agent soman [18].

In experimental models of SE induced by OP nerve agents, the early neuroinflammatory responses typically coincide with neurodegeneration (reviewed in [3–5, 12]). Similarly, we detected significant neurodegeneration during the first 3 days post-exposure in multiple brain regions, including the hippocampus and cortex, as evidenced by an increased number of FJB-labeled cells, which we previously identified as neurons [35]. In all the brain regions examined, the number of FJB-labeled neurons remained elevated at 7 and 14 days post-exposure but decreased to control levels by 21 days post-exposure. In the hippocampus, neurodegeneration coincided with reactive astrogliosis but ended prior to the decrease in microglial cell activation. In contrast, in the cortex, reactive astrogliosis persisted beyond the period of neurodegeneration, while microglial cell activation returned to control levels prior to the end of the neurodegenerative period. These observations raise interesting questions regarding the relationship between neuroinflammation and neurodegeneration in acute DFP intoxication. In previous studies of this model, we observed significantly increased GFAP immunoreactivity in the piriform cortex, amygdala, and dentate gyrus at 1 h post-DFP exposure [31], at which time we saw no evidence of neuronal cell death in these brain regions [35], suggesting that the initial activation of astrocytes occurs independent of neurodegeneration. In experimental models of acute soman intoxication, microglial activation follows an early increase in GFAP immunoreactivity and precedes neuronal injury, suggesting that soman-induced SE induces astrogliosis resulting in a neuroinflammatory response that contributes to neuronal injury [16, 52]. However, a study suggesting that delayed astrogliosis mediates synaptic plasticity and neuronal repair in mice acutely intoxicated with soman [53] supports the model of a dynamic relationship between neurodegeneration and neuroinflammation that changes over time.

Persistent memory impairment is one of the most common behavioral consequences observed in humans who survive acute OP poisoning [5]. Years after the 1995 Tokyo subway attack with sarin, exposed rescue personnel and subway workers exhibited significant deficits on memory tests compared to non-exposed control subjects [54, 55]. OP-induced SE has also been shown to impair memory in preclinical models [38, 39, 56, 57]. Consistent with these reports, we observed that DFP-induced SE significantly impaired performance in contextual fear conditioning at 9 and 20 days post-exposure. These data corroborate a recent publication reporting that rats intoxicated with DFP, using the same dosing paradigm used here, exhibited impaired performance in the novel object recognition task [39]. Surprisingly, however, cognitive deficits in DFP-intoxicated rats did not vary according to TSPO phenotype or seizure severity score. Specifically, a subset of DFP-intoxicated animals with moderate seizure severity scores and either minimal or early, but non-persistent, TSPO labeling as determined by PET imaging exhibited deficits in the contextual fear-conditioning task that were comparable to those of DFP-intoxicated animals with high seizure severity scores and significant TSPO labeling at all three post-exposure imaging times. This observation suggests that acute DFP intoxication causes cognitive deficits independent of seizure activity and neuroinflammation. Alternative mechanisms of OP neurotoxicity that may be relevant include cholinergic excitotoxicity [5], oxidative stress [50, 58], and impaired microtubule function [59].

While our data suggest that neither seizure severity nor neuroinflammation is the primary determinant of DFP-induced cognitive deficits, several important caveats support the need for further studies to confirm this interpretation. First, since the animals could not be instrumented to collect electrographic data, we cannot rule out the possibility that the subset of animals with the lower maximal seizure severity scores experienced seizures of longer duration such that their “average” seizure score over time may be equivalent to that of the animals with the higher maximal seizure severity scores. If this were the case, based on data indicating that the duration of seizure activity is a predominant determinant of pathological outcome following acute intoxication with OP nerve agents [11], it would not be surprising that the two groups of DFP animals exhibited comparable deficits in cognitive behavior. Alternatively, it may be that the influence of seizure activity on cognitive behavior is not a linear correlation in which seizures of greater intensity and/or duration cause proportionately more severe behavioral deficits, but rather, there is a threshold level of neural damage required to elicit behavioral deficits. In this case, the moderate seizure behavior experienced by the DFP animals exhibiting the alternative TSPO phenotype may have generated sufficient neural damage to cross this threshold. Second, because PET imaging was only performed at 2, 7, and 21 days post-exposure, it is possible that we missed an increase in TSPO labeling that occurred between these imaging days in the group of animals with the alternate TSPO phenotype. Third, because the PET imaging and immunocytochemical studies of neuroinflammation were performed in separate cohorts of animals, we cannot determine the extent to which [^11^C]-(R)-PK11195 labels activated microglia vs. reactive astrocytes. This may be an important consideration in light of a recent report that an inhibitor of the prostaglandin E2 receptor EP2, which significantly decreases microglial activation but not reactive astrogliosis during the first few days following acute intoxication with DFP [34], was also effective in attenuating deficits in the novel object recognition task [39]. These findings suggest that microglial activation, but not reactive astrogliosis, is causally linked to memory impairment following OP-induced SE. This remains to be proven; however, if true, it will be important to determine whether the TSPO signal in our PET imaging data is due predominantly to binding of [^11^C]-(R)-PK11195 by astrocytes or microglia. If the PET signal is driven primarily by the astrocytic expression of TSPO, PET imaging of [^11^C]-(R)-PK11195 binding may have missed more subtle, but functionally significant, increases in microglial activation. It will also be important to confirm our semi-quantitative assessments of reactive astrogliosis and microglial activation by immunocytochemistry and PET imaging using western blotting or ELISA. Last, a key feature of altered cognition associated with neuroinflammation is the altered expression of proinflammatory cytokines and chemokines. These factors were not examined in the present study; thus, the observed lack of correlation between GFAP and IBA1 immunoreactivity with impaired performance in contextual fear conditioning does not definitively rule out a role for neuroinflammation in the pathogenesis of DFP-induced cognitive deficits.

## Conclusions

We describe a preclinical model in which acute DFP intoxication causes seizures, persistent neuroinflammation, and cognitive deficits. The extent of the neuroinflammatory response is influenced by seizure severity. However, the observation that a subset of animals with moderate seizures and minimal TSPO labeling exhibited cognitive deficits comparable to those of animals with severe seizures and significant TSPO labeling raises questions as to the role of both seizures and neuroinflammation in the pathogenesis of cognitive dysfunction following acute OP intoxication. Collectively, these findings, together with previous reports suggesting that microglial activation, but not reactive astrogliosis, is linked to DFP-induced memory impairment [34, 39] and that delayed astrogliosis enhances neurogenesis and accelerates functional recovery of affective, but not cognitive, behavior [53], suggest that the role of neuroinflammation likely changes post-exposure as the profile of neuroinflammatory cell activation evolves. Further, these observations suggest that the influence of neuroinflammation is likely to vary across the diverse neurological outcomes associated with acute OP intoxication.

## Abbreviations

DFP: Diisopropylfluorophosphate
FJB: FluoroJade-B
GFAP: Glial fibrillary acidic protein
IBA1: Ionized calcium-binding adapter molecule 1
OP: Organophosphorus
PET: Positron emission tomography
SE: Status epilepticus
TSPO: Translocator protein
